# Acetabular posterior column screws via an anterior approach

**DOI:** 10.1007/s00402-024-05471-7

**Published:** 2024-08-07

**Authors:** Dietmar Krappinger, Axel Gänsslen, Lukas Wilde, Richard A. Lindtner

**Affiliations:** 1 Department of Orthopaedics and Traumatology, Medical University of Innsbruck, Innsbruck, Austria; 2 Department of Trauma Surgery, Orthopaedics and Hand Surgery, Wolfsburg General Hospital, Wolfsburg, Germany

**Keywords:** Acetabulum, Acetabular fracture, Internal fixation, Anterior column and posterior hemitransverse fracture, Posterior column screw, Malreduction, Hip joint penetration, Cortical perforation

## Abstract

Screw fixation of acetabular column fractures is a well-established alternative option to plate fixation providing comparable biomechanical strength and requiring less surgical exposure. For displaced acetabular fractures involving both columns open reduction and plate fixation of one column in combination with a column-crossing screw fixation of the opposite column via a single approach is a viable treatment option. Preoperative planning of posterior column screws (PCS) via an anterior approach is mandatory to assess the eligibility of the fracture for this technique and to plan the entry point and the screw trajectory. The intraoperative application requires fluoroscopic guidance using several views. A single view showing an extraarticular screw position is adequate to rule out hip joint penetration. The fluoroscopic assessment of cortical perforation of the posterior column requires several oblique views such as lateral oblique views, obturator oblique views and axial views of the posterior column or alternatively intraoperative CT scans. The application of PCS via an anterior approach is a technically demanding procedure, that allows for a relevant reduction of approach-related morbidity, surgical time and blood loss by using a single approach.

## Rationale of acetabular column screw fixation

Screw fixation of acetabular column fractures was first described by Letournel and Judet in the 1960s [1]. It is nowadays a well-established alternative option to plate fixation with the advantage of requiring less surgical exposure [2–7]. Several biomechanical studies and finite element analyses have shown that screw fixation of acetabular column fractures provides mechanical strength comparable to plate fixation [8–17].

Percutaneous screw fixation is therefore a viable minimally invasive option for the fixation of non- or minimally displaced acetabular column fractures. The screws may be inserted in an antegrade or retrograde manner into the anterior or posterior column [3, 6, 16, 18–23]. The main complication of these techniques is screw misplacement resulting either in (a) hip joint penetration or (b) cortical perforation of the acetabular column. The latter may result in damage of surrounding neurovascular structures. Accordingly, high-quality intraoperative imaging is of utmost importance to prevent these complications. Several radiographic views such as the inlet-iliac view, the outlet-obturator view, the obturator oblique view and axial views of the acetabular columns are described in the literature for percutaneous screw fixation of acetabular column fractures under fluoroscopic guidance [6, 18, 21–24].

Displaced acetabular fractures are not suitable for percutaneous fixation techniques. Internal fixation of these fractures requires open reduction of the fracture prior to fixation. It is well-known that the quality of reduction is one of the most important outcome parameters in acetabular fracture surgery [25–27]. For example, displaced posterior wall and / or column fractures require a posterior approach, while displaced anterior column fractures are reduced via an anterior approach. It is also known from several biomechanical studies that fractures involving both columns require internal fixation of both columns to achieve sufficient biomechanical stability for immediate postoperative mobilization [4, 8–11, 14, 17]. Accordingly, plate fixation of both columns may appear desirable from a biomechanical point of view but requires a combined anterior and posterior approach and thus is associated with increased surgical time, blood loss and approach-related morbidity.

A clinically more appealing option for the internal fixation of displaced acetabular fractures involving both columns is open reduction and plate fixation of one column in combination with a column-crossing screw fixation of the opposite column via a single approach, i.e. antegrade posterior column screws (PCS) via an anterior approach or antegrade anterior column screws (ACS) via a posterior approach. These techniques allow for a reduction of the approach-related morbidity by obviating the need for a second surgical approach [7, 28–31]. Screw misplacement resulting either in (a) hip joint penetration or (b) cortical perforation of the acetabular column represent the main complication of these techniques. Additionally, inadequate reduction of the opposite column fracture is a potential drawback as well [5]. Accordingly, thorough preoperative (a) fracture analysis and (b) osseous corridor analysis as well as (c) intraoperative imaging using appropriate views are mandatory.

ACS via a posterior approach are less frequently used than PCS via an anterior approach [5, 21, 29]. Demographic changes towards the elderly with an increasing involvement rate of the anterior structures of the acetabulum requiring an anterior approach may account for this finding [32, 33]. Additionally, ACS are technically more demanding due to the smaller diameter of the anterior column [8, 32]. Liu et al. reported a series of 12 patients with transverse fractures treated with posterior plating and ACS compared to a group of 17 patients with combined anterior and posterior plating [29]. The application of ACS resulted in less surgical time, blood loss and invasiveness. The reduction of the anterior column fracture was performed using threaded pins in the ischial tuberosity as joysticks and clamps with palpation of the anterior column fracture through the greater sciatic notch (aka „the endopelvic finger“ [32]. Hammad et al. reported a series of 34 patients with T-type fractures treated with posterior plating and ACS [5]. The authors stated that the limited access to the anterior column is a major issue of this technique. Accordingly, they found a residual displacement of the anterior column in 12 cases (32%), which negatively affected the hip joint congruency and consecutively the clinical outcomes. These results clearly demonstrate the importance of the reduction of the opposite column when using either column-crossing ACS or PCS.

The infraacetabular screw (IAS) was initially described by Letournel and Judet (“long screws parallel to the quadrilateral surface and crossing the fracture line”) [34] and gained broader recognition and usage thanks to the work of Culemann et al. in 2011 [4]. By definition, it is a column-crossing screw as well. The IAS is inserted via an anterior approach and ends in the posterior column. IAS increases the stability of the fixation by closing the periacetabular fixation frame without requiring an additional approach [4, 11, 14, 17, 33, 35]. The major drawback of this technique is the narrow osseous screw corridor with close proximity to the hip joint [36]. Depending on the depth of the acetabular fossa, the screw path may even be partially intraarticular [4, 37]. Gras et al. showed that there was no osseous corridor with a diameter of more than 5 mm in 7% of the patients [38]. This rate is even higher in female patients [35]. Additionally, the IAS does not cross the posterior column fracture and therefore does not act as a lag screw in transverse fractures and anterior column and posterior hemitransverse (ACPHT) fractures. In contrast, PCS cross the posterior fracture lines nearly perpendicular and may act as lag screws in non-comminuted posterior column fractures. Accordingly, PCS provided superior biomechanical stability compared to IAS for posterior column fractures in a recent biomechanical study [17]. Obviously, it is also possible to combine IAS and PCS for additional mechanical stability as well.

The preoperative planning as well as the intraoperative application and complications of PCS via an anterior approach are described in the following sections.

## Anatomy of the posterior column screw corridor

The osseous corridor for antegrade screw placement starts in the iliac fossa close to the SI joint and then runs into the entire posterior column to the ischial tuberosity. Relevant surrounding anatomical structures include the joint itself and the sciatic nerve, the latter is close to the ischial tuberosity [39].

Letournel defined the antegrade entry point to be 1 cm anterior to the SI joint in direction to the pelvic brim and 25 mm lateral and perpendicular to the pelvic brim [34]. Mu et al. defined the entry point by two lines: the first line started at the anterior SI joint along the pelvic brim, and the second line was orientated perpendicular and medial to the first line reaching the entry point. The first line had a length of 23.5 mm on average and the second line a length of 16.8 mm [2]. The distance between the entry point for an antegrade PCS and tangential to the anterior border of the SI joint was 32–35 mm [40].

CT-based or cadaver measurements analyzed the length of the PCS-Corridor. A mean length of approximately 105 mm is reported [2, 41, 42] without side differences [43].

Knowledge of angulations of the drill bit may help to determine the optimal screw course. An angle between the screw and the sagittal (AP view, medial orientation) of 18° and coronal planes (lateral view, retroversion) of 12–15° was reported in CT based studies [30, 41], while the angle between the quadrilateral surface and the iliac wing plane was 132.3° [30].

## Preoperative planning of PCS

The preoperative planning of PCS comprises both a fracture analysis and an osseous corridor analysis. The first step in the preoperative planning of PCS therefore is the assessment of the eligibility of the acetabular fracture for using PCS via an anterior approach. The following fracture types involve both columns:

- Transverse fractures (± posterior wall).
- Anterior column and posterior hemitransverse (ACPHT) fractures.
- T-type fractures.
- Associated both column fractures (± posterior wall).

A posterior approach is required if there is an indication for internal fixation of the posterior wall fracture. Accordingly, these fracture types are not eligible for PCS. A major prerequisite for successful application of PCS is adequate reduction of the posterior column fracture via the anterior approach. Transverse fractures comprise a single fracture line, which allows for reduction of the posterior hemitransverse fracture via the anterior approach.

**Fig. 1:**
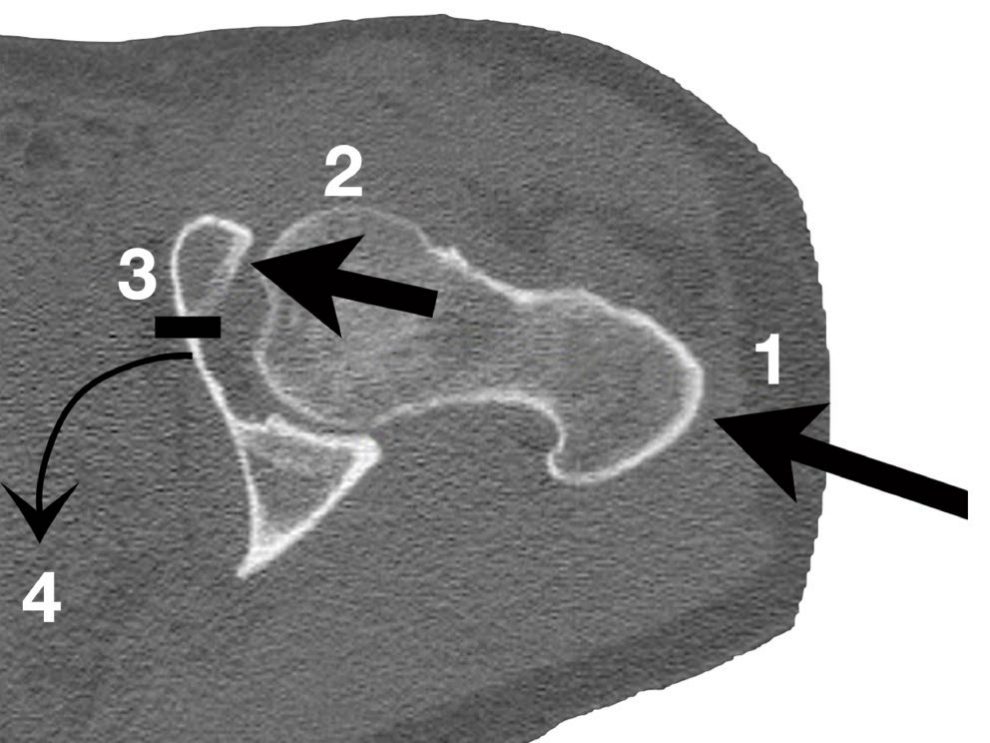
Injury mechanism of ACPHT fractures: (1) Force transmission via the greater trochanter. (2) Direction of the force vector to the anterior and superior part of the acetabulum. (3) Fracture between the anterior column and the medial wall. (4) Posterior hemitransverse fracture and internal rotation of the posterior column

ACPHT fractures represent a relatively uniform fracture pattern despite their cumbersome name. ACPHT fractures typically result from force transmission via the greater trochanter and the femoral neck with the hip joint in extension (1 in Fig. 1). Due to the anteversion of the femoral neck the anterior column is affected first and frequently shows a displaced multifragmentary fracture pattern (2 in Fig. 1). Onward force transmission leads to a fracture between the anterior column and the medial wall (3 in Fig. 1). Additionally, the eponymous hemitransverse fracture of the posterior column typically shows a simple fracture pattern and allows for an internal rotation of the posterior column (4 in Fig. 1). Accordingly, the medial wall is not „medialized“ or „centralized“ in its entirety following a translational displacement, as stated by several authors. Instead, the medial wall is in osseous continuity with the posterior column and shows a rotational displacement due to the internal rotation of the posterior column. Accordingly, it is feasible to reduce the posterior hemitransverse fracture component by reducing the rotational displacement of the medial wall via an anterior approach.

Associated both column fractures represent a relatively heterogenous fracture type. This fracture type is defined by a separation of both columns from each other combined with a separation of the acetabulum from the residual iliac bone and the sacroiliac joint (aka „floating acetabulum“, [32]). Some associated both column fractures share fracture characteristics with ACPHT fractures and are therefore reducible via the medial wall as described above. The posterior fracture component of other both-column fractures may be indirectly reduced via a secondary congruency. However, a recommendation for or against the application of PCS in both-column fractures needs to be made for each particular case. The same is true for T-type fractures.

The second step in the preoperative planning of PCS is the assessment of the entry point and the screw trajectory. This is especially important due to the heterogeneity of the osseous corridor [7, 44, 45] based on interindividual differences, gender differences and also differences between patients with and without sacral dysmorphism [44]. In general, both an entry point at the inner cortex of the iliac bone in the transition zone between the supraacetabular region and the iliac wing [2, 46, 47] and as well as an entry point at the iliac crest [15, 31] are feasible. The use of custom-made guide templates based on the preoperative planning may facilitate the intraoperative application of PCS [15, 30, 48].

**Fig. 2:**
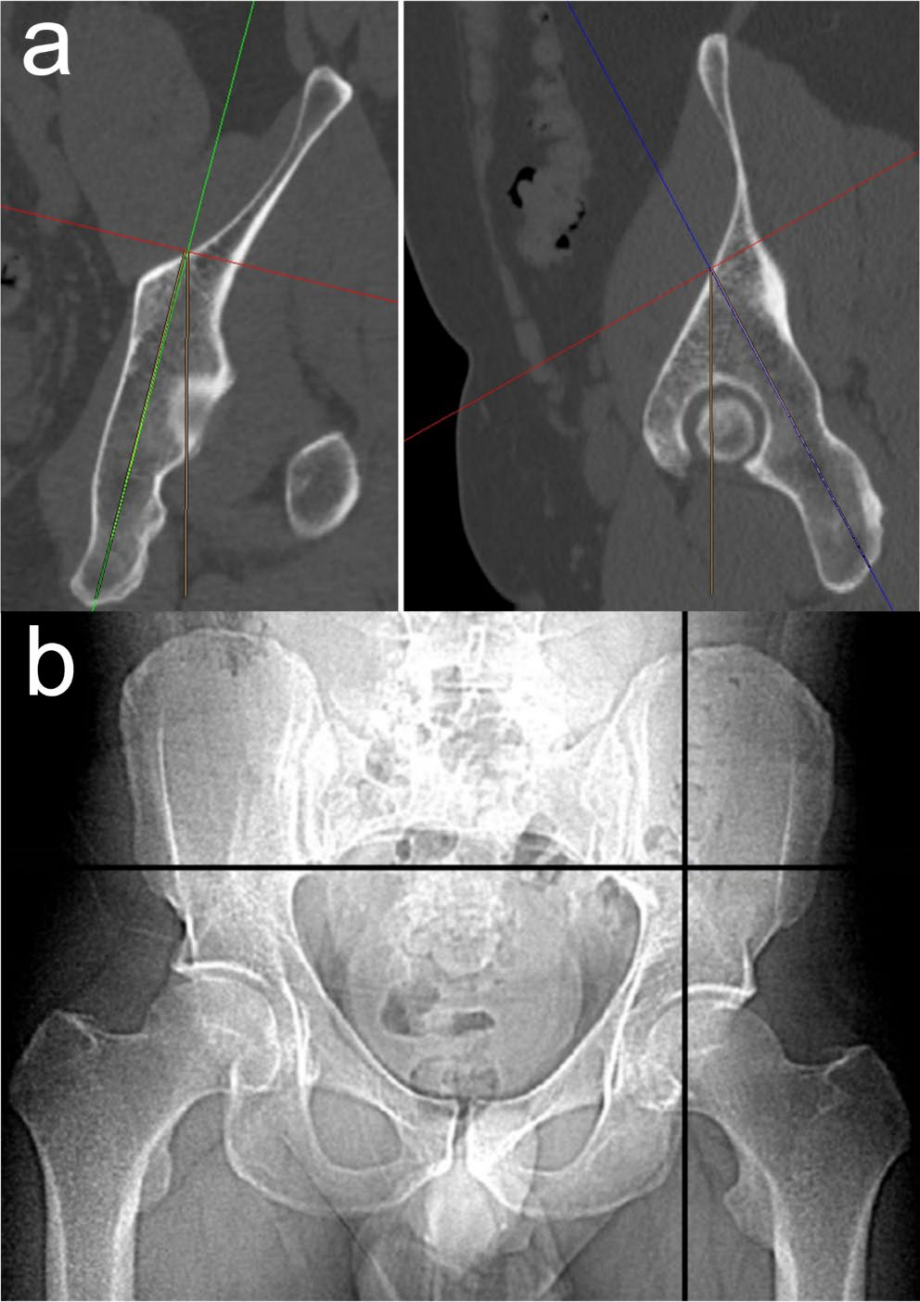
PCS entry point and screw trajectory. **a** Preoperative planning of the entry point and the screw trajectory of PCS using standard pelvic CT scans and commonly available multiplanar reconstruction tools. The entry point is located at the inner cortex of the iliac bone in the transition zone between the supraacetabular region and the iliac wing. The screw trajectory is typically oriented from cranial–anterior–lateral to caudal–posterior–medial. Coronal reconstruction (left) and sagittal reconstruction (right). **b** Determination of the entry point using the LiveSync feature in the CT scout image. The CT scout image is performed in a standard supine position and thus corresponds to the intraoperative positioning of the patient

The authors´ approach is to use a standard pelvic CT scan and commonly available multiplanar reconstruction tools. In the presence of severe fracture displacement, it is advisable to perform the preoperative planning on the contralateral side [47]. We choose an entry point at the inner cortex of the iliac bone in the transition zone between the supraacetabular region and the iliac wing. The screw trajectory is oriented from cranial–anterior–lateral to caudal–posterior–medial. Using a two-dimensional multiplanar software reconstruction tool, the axes of coordinates are translated and the axes itself rotated to assess the ideal entry point and screw trajectory (Fig. 2a). The preoperative planning is performed using multiplanar CT reconstructions in all three dimensions. The intraoperative application, however, is generally performed using two-dimensional (2D) fluoroscopic control. The CT scout image is therefore used to facilitate this transition. The CT scout image is usually performed in a standard supine position and thus corresponds to the intraoperative positioning of the patient. Software tools allow for a real-time localization of any arbitrary CT point in the scout view (“LiveSync” feature). Thus, the surgeon is able to transfer the ideal entry point identified in 3D CT reconstructions to the 2D scout image, which corresponds to the intraoperative pelvic ap view (Fig. 2b).

## Fluoroscopic projections PCS

For posterior column screws, all possible standard views and their combinations were recommended including the standard pelvic ap view, pelvic inlet view (PIV), obturator oblique view (OOV), iliac oblique view IOV, true lateral view (LV), combined obturator oblique outlet view (COOO), combined iliac oblique outlet (CIOO) view and a half CIOO [11, 12, 49–51]. Bishop et al. recommended the IOV, the pelvic outlet view and the true LV [52].

The IOV defines the posterior border of the corridor, visualizing the sciatic notches and the sciatic spine; this view confirms the screw position posterior to the hip joint. The PIV confirms the localization of the medial border (quadrilateral surface). The true LV analyzes the pelvic brim and confirms the screw exit on the inner table of the iliac fossa.

Additionally, the OOV confirms the screw position at the distal ischium.

## Intraoperative application of PCS

The intraoperative application of PCS is based on the preoperative planning using cannulated large fragment screws. There are two major prerequisites for a successful application, which need to be ruled out intraoperatively, i.e. (a) hip joint penetration and (b) cortical perforation of the posterior column. In general, ruling out hip joint penetration is not dependent on a particular fluoroscopic view. It was shown in a recent study that in a concave surface such as the acetabulum a single and arbitrary view showing an extraarticular screw position is adequate to rule out hip joint penetration [53]. The standard fluoroscopic views used for PCS are an ap view, an iliac oblique view and an obturator oblique view [22, 24]. Osterhoff et al. stated that these standard views are not able to rule out medial cortical perforation of the posterior column due to the saddle-shaped curvature of the medial cortex [24]. Accordingly, a „Down to PC“ view tangentially to the medial cortex and in the desired axis of the PCS was proposed.

**Fig. 3:**
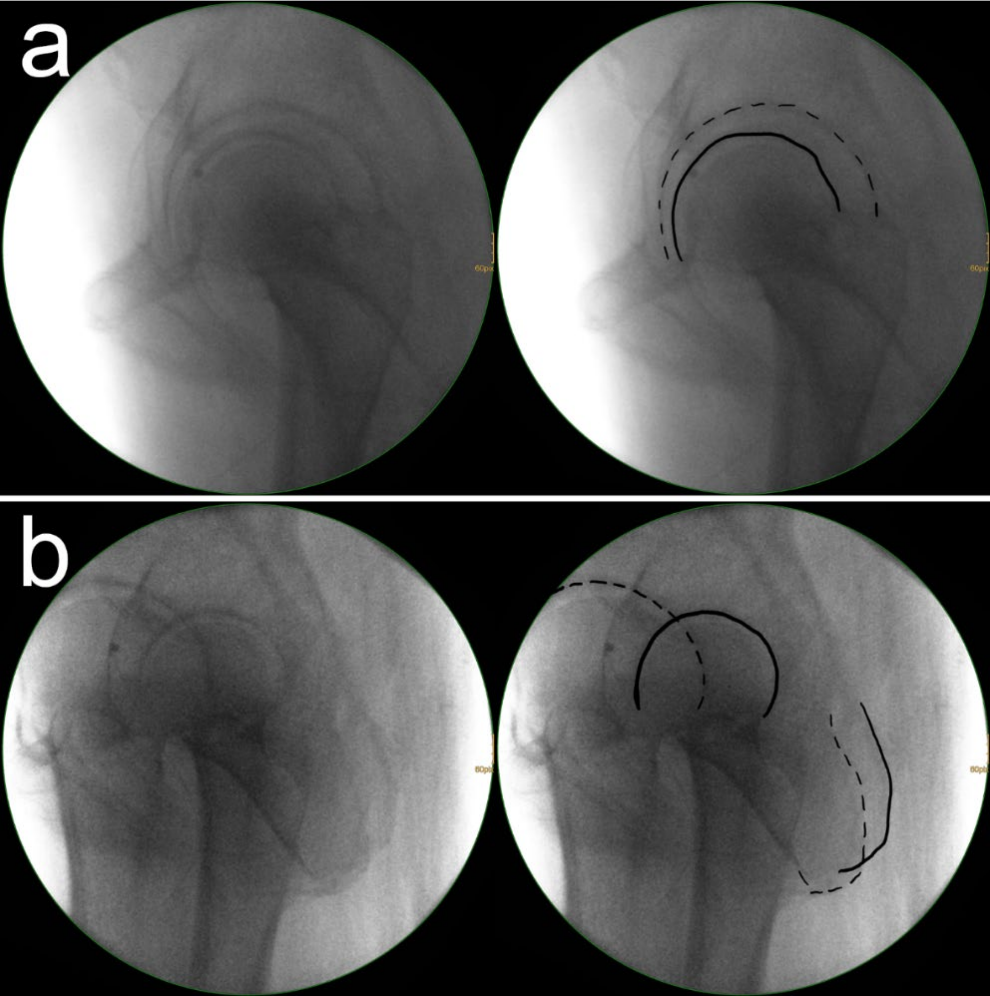
**a** Lateral view of the hip. The “larger” hip (dotted line) is located near the radiation source, while the “smaller” hip (solid line) is located near the receiver of the C-arm. This allows for an easy side differentiation using the magnification effect. **b** The lateral oblique view is obtained by tilting down the C-arm approximately 15° on the involved side in order to have a fluoroscopic view of the posterior border of the involved posterior column (solid line)

The authors´ approach is to use a standard ap view and a lateral oblique view [47]. The ap view allows for the assessment of the entry point according to preoperative planning and the screw trajectory in the coronal plane. The lateral oblique view allows for the assessment of the screw trajectory in the sagittal plane and for ruling out hip joint penetration. In general, the lateral view of the hip is not very commonly used, as it is inevitable that both hips are projected into each other. The magnification effect, however, allows for a simple differentiation between the two hips. The “larger” hip (Fig. 3a, dotted line) is located near the radiation source, while the “smaller” hip (Fig. 3a, solid line) is located near the receiver of the C-arm. The lateral oblique view is obtained by starting with a lateral view and tilting down the C-arm approximately 15° on the involved side in order to have a fluoroscopic view of the posterior border of the posterior column (Fig. 3b, solid line). It is advisable to internally rotate the legs to prevent fluoroscopic projection of the femoral neck and the greater trochanter onto the posterior border of the posterior column.

**Fig. 4:**
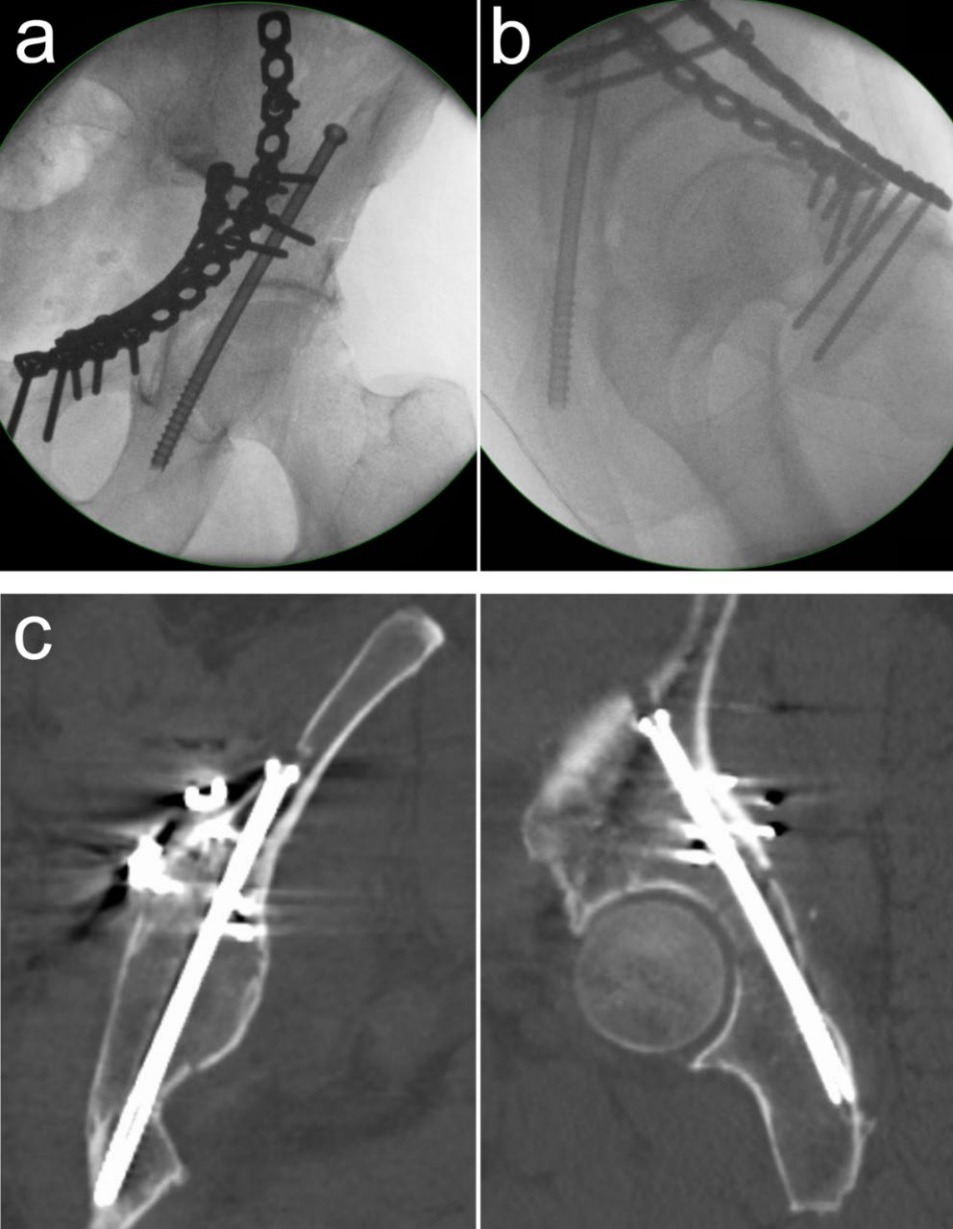
Correct screw placement **a** Intraoperative ap view of an ACPHT fracture treated with open reduction and plate fixation of the anterior column and PCS for the fixation of the posterior hemitransverse fracture. The ap view shows the correct screw trajectory in the coronal plane. **b** The lateral oblique view shows the correct screw trajectory in the sagittal plane as well as an extraarticular screw trajectory with no perforation of the posterior cortex of the posterior column. **c** The postoperative CT scan confirms the intraoperative findings. The posterior hemitransverse fracture is anatomically reduced. Coronal reconstruction (left) and sagittal reconstruction (right)

Figure 4 shows an ACPHT fracture treated with open reduction and plate fixation of the anterior column and PCS for the posterior hemitransverse fracture component including an intraoperative ap view (Fig. 4a), an intraoperative lateral oblique view (Fig. 4b) and a postoperative CT scan (Fig. 4c).

## Complications of PCS

Between July 2016 and June 2022, 27 patients (20 men and 7 women) were treated with PCS via an anterior approach at the first author´s institution. There were 14 ACPHT fractures, 10 both-column fractures and 3 transverse fractures. Postoperative CT scans showed residual displacement of the posterior column of < 2 mm in 21 cases and between 2 and 5 mm in 6 cases. There was one hip joint penetration and three cortical perforations of the posterior column without neurovascular deficits. These complications are discussed in the following two paragraphs.

### Hip joint penetration

**Fig. 5:**
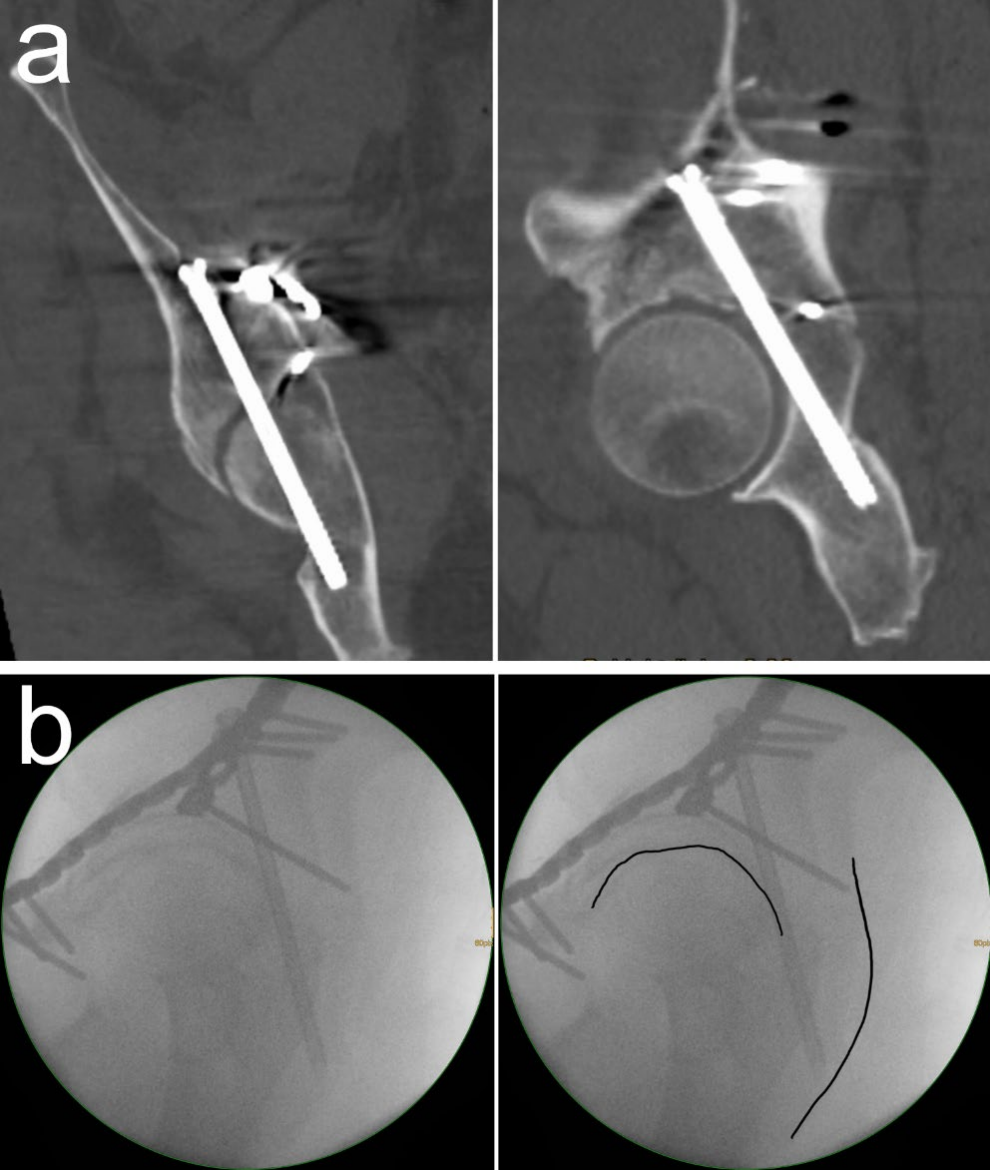
Hip joint penetration. **a** Postoperative CT scan of an ACPHT fracture treated with PCS showing adequate reduction of the posterior hemitransverse fracture and no posterior cortical perforation of the posterior column, but hip joint penetration. Coronal reconstruction (left) and sagittal reconstruction (right). **b** The lateral oblique view shows the close proximity of the PCS to the hip joint despite poor image quality and poor side differentiation

In general, ruling out hip joint penetration simply requires a single view showing an extraarticular screw position [53]. The authors´ approach is to use the lateral oblique view. Figure 5a shows a postoperative CT scan of a PCS with adequate reduction of the posterior hemitransverse fracture and no posterior cortical perforation of the posterior column, but with hip joint penetration. Figure 5b shows the corresponding intraoperative lateral oblique view with poor visualization of the anatomical landmarks. Additionally, the lateral oblique view was not sufficiently oblique resulting in a poor side differentiation. Despite these fluoroscopic limitations the proximity of the PCS to the hip joint is clearly visible (left side). This complication therefore results from an intraoperative error. Postoperatively, the hip joint motion was not restricted, and the patient refused revision surgery.

### Cortical perforation of the posterior column

**Fig. 6:**
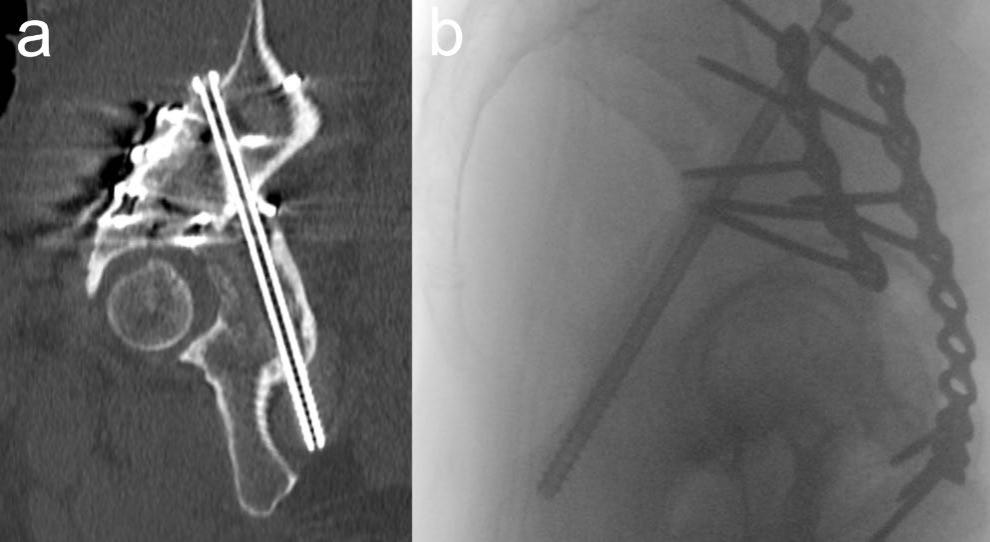
Cortical perforation of the posterior column. **a** Postoperative CT scan of an ACPHT fracture treated with PCS showing adequate reduction of the posterior hemitransverse fracture and no hip joint penetration, but posterior cortical perforation of the posterior column. Sagittal reconstruction. **b** The posterior cortical perforation is not clearly visible in a intraoperative lateral oblique view

Ruling out cortical perforation of the posterior column using fluoroscopy is demanding due to the narrow and heterogenous anatomy of the posterior column, which was described as hyperbolic paraboloid [24] and triangular-prism shaped [22]. The authors´ approach is to use the lateral oblique view as well. Figure 6a shows a postoperative CT scan of a PCS with adequate reduction of the posterior hemitransverse fracture and no hip joint penetration, but with posterior cortical perforation of the posterior column. Figure 6b shows the corresponding intraoperative lateral oblique view, which does not clearly show cortical perforation despite a relatively posterior screw trajectory. Accordingly, additional views such as the Down-to-PC view [24] or iliac oblique views [22] may be mandatory in order to rule out cortical perforation under fluoroscopic guidance [7]. Postoperatively, there was no sciatic nerve palsy and no problems with sitting in this case.

## Conclusion

The application of PCS via an anterior approach is a technically demanding procedure, that allows for a relevant reduction of approach-related morbidity, surgical time and blood loss by using a single approach. Potential risks of this technique are inadequate reduction of the posterior column as well as screw misplacement with hip joint penetration and cortical perforation of the posterior column. It therefore requires meticulous preoperative planning and accurate intraoperative fluoroscopic guidance.

## References

[1] Letournel E (1966) [Surgical management of hip joint-acetabulum fractures]. Langenbecks Arch Chir 316:422–437 5979710 10.1007/BF02433653

[2] Mu WD, Wang XQ, Jia TH, Zhou DS, Cheng AX (2009) Quantitative anatomic basis of antegrade lag screw placement in posterior column of acetabulum. Arch Orthop Trauma Surg 129(11):1531–1537 19221771 10.1007/s00402-009-0836-6

[3] Chen KN, Wang G, Cao LG, Zhang MC (2009) Differences of percutaneous retrograde screw fixation of anterior column acetabular fractures between male and female: a study of 164 virtual three-dimensional models. Injury 40(10):1067–1072 19329113 10.1016/j.injury.2009.01.014

[4] Culemann U, Marintschev I, Gras F, Pohlemann T (2011) Infra-acetabular corridor–technical tip for an additional screw placement to increase the fixation strength of acetabular fractures. J Trauma 70(1):244–246 21217495 10.1097/TA.0b013e3181f45f91

[5] Hammad AS, El-Khadrawe TA, Waly AH, Abu-Sheasha GA (2017) The efficacy of posterior plating and anterior column screw fixation in the management of T-shaped acetabular fractures - CART analysis of prospective cohort study. Injury 48(3):680–686 28104228 10.1016/j.injury.2017.01.024

[6] Guimarães JAM, Martin MP 3rd, da Silva FR et al (2019) The obturator oblique and iliac oblique/outlet views predict most accurately the adequate position of an anterior column acetabular screw. Int Orthop 43(5):1205–1213 10.1007/s00264-018-3989-5 29948010

[7] Cavalié G, Boudissa M, Kerschbaumer G, Seurat O, Ruatti S, Tonetti J (2022) Clinical and radiological outcomes of antegrade posterior column screw fixation of the acetabulum. Orthop Traumatol Surg Res 108(4):103288 35470116 10.1016/j.otsr.2022.103288

[8] Le Quang H, Schmoelz W, Lindtner RA, Schwendinger P, Blauth M, Krappinger D (2020) Biomechanical comparison of fixation techniques for transverse acetabular fractures - single-leg stance vs. sit-to-stand loading. Injury 51(10):2158–2164 32646647 10.1016/j.injury.2020.07.008

[9] Le Quang H, Schmoelz W, Lindtner RA, Dammerer D, Schwendinger P, Krappinger D (2021) Single column plate plus other column lag screw fixation vs. both column plate fixation for anterior column with posterior hemitransverse acetabular fractures - a biomechanical analysis using different loading protocols. Injury 52(4):699–704 33454060 10.1016/j.injury.2020.12.041

[10] Khajavi K, Lee AT, Lindsey DP, Leucht P, Bellino MJ, Giori NJ (2010) Single column locking plate fixation is inadequate in two column acetabular fractures. A biomechanical analysis. J Orthop Surg Res 5:30 20459688 10.1186/1749-799X-5-30 PMC2876138

[11] Gras F, Marintschev I, Schwarz CE, Hofmann GO, Pohlemann T, Culemann U (2012) Screw- versus plate-fixation strength of acetabular anterior column fractures: a biomechanical study. J Trauma Acute Care Surg 72(6):1664–1670 22695438 10.1097/TA.0b013e3182463b45

[12] Becker CA, Kammerlander C, Cavalcanti Kußmaul A et al (2018) Minimally invasive screw fixation is as stable as anterior plating in acetabular T-Type fractures - a biomechanical study. Orthop Traumatol Surg Res 104(7):1055–1061 30179721 10.1016/j.otsr.2018.06.013

[13] Busuttil T, Teuben M, Pfeifer R, Cinelli P, Pape HC, Osterhoff G (2019) Screw fixation of ACPHT acetabular fractures offers sufficient biomechanical stability when compared to standard buttress plate fixation. BMC Musculoskelet Disord 20(1):39 30678661 10.1186/s12891-019-2422-6 PMC6346571

[14] Wang T, Zhao B, Yan J, Shao B, Mu W (2022) Finite element analysis of infra-acetabular screw fixation for the treatment of acetabular posterior column fracture. Int Orthop 46(3):623–634 34981159 10.1007/s00264-021-05298-6

[15] Ye J, Xie L, Liu Z, Lin J, Yan H, Chen Z (2022) Anterograde lag screw placement in the posterior column of the acetabulum: a case report and literature review. Trauma Case Rep 37:100580 34917743 10.1016/j.tcr.2021.100580 PMC8669443

[16] Berk T, Zderic I, Schwarzenberg P et al (2023) Simulated full weight bearing following posterior column acetabular fracture fixation: a biomechanical comparability study. J Orthop Surg Res 18(1):401 37268974 10.1186/s13018-023-03879-2 PMC10236575

[17] Hinz N, Baumeister D, Dehoust J et al (2024) The infraacetabular screw versus the antegrade posterior column screw in acetabulum fractures with posterior column involvement: a biomechanical comparison. Arch Orthop Trauma Surg 10.1007/s00402-024-05324-3 PMC11211174 38676740

[18] Zheng Z, Wu W, Yu X et al (2015) Axial view of acetabular anterior column: a new X-ray projection of percutaneous screw placement. Arch Orthop Trauma Surg 135(2):187–192 25450306 10.1007/s00402-014-2127-0

[19] Feng X, Fang J, Lin C et al (2015) Axial perspective to find the largest intraosseous space available for percutaneous screw fixation of fractures of the acetabular anterior column. Int J Comput Assist Radiol Surg 10(8):1347–1353 25572704 10.1007/s11548-015-1149-6

[20] Peng Y, Zhang L, Min W, Tang P (2016) Comparison of anterograde versus retrograde percutaneous screw fixation of anterior column acetabular fractures. Int J Comput Assist Radiol Surg 11(4):635–639 26476636 10.1007/s11548-015-1308-9

[21] Wang X, Ran G, Chen X et al (2021) Obturator Oblique and Pubic Ramus Inlet views can Better Guide the insertion of an Anterior Column Acetabular Screw. Orthop Surg 13(3):1086–1093 33821566 10.1111/os.12943 PMC8126927

[22] Yu K, Zhou R, Gao S, Liang A, Yang M, Yang H (2022) The placement of percutaneous retrograde acetabular posterior column screw based on imaging anatomical study of acetabular posterior column corridor. J Orthop Surg Res 17(1):492 36384572 10.1186/s13018-022-03347-3 PMC9670384

[23] Cattaneo S, Galante C, Biancardi E et al (2023) Use of the iliac-outlet and iliac-inlet combined views in percutaneous posterior column retrograde screw fixation. Arch Orthop Trauma Surg 143(9):5713–5717 37284880 10.1007/s00402-023-04939-2 PMC10449713

[24] Osterhoff G, Amiri S, Unno F et al (2015) The Down the PC view - a new tool to assess screw positioning in the posterior column of the acetabulum. Injury 46(8):1625–1628 25990076 10.1016/j.injury.2015.04.042

[25] Matta JM (1996) Fractures of the acetabulum: accuracy of reduction and clinical results in patients managed operatively within three weeks after the injury. J Bone Joint Surg Am 78(11):1632–1645 8934477

[26] Tannast M, Najibi S, Matta JM (2012) Two to twenty-year survivorship of the hip in 810 patients with operatively treated acetabular fractures. J Bone Joint Surg Am 94(17):1559–1567 22992846 10.2106/JBJS.K.00444

[27] Verbeek DO, van der List JP, Tissue CM, Helfet DL (2018) Predictors for long-term hip survivorship following Acetabular fracture surgery: importance of gap compared with step displacement. J Bone Joint Surg Am 100(11):922–929 29870442 10.2106/JBJS.17.00692

[28] Jung GH, Lee Y, Kim JW, Kim JW (2017) Computational analysis of the safe zone for the antegrade lag screw in posterior column fixation with the anterior approach in acetabular fracture: a cadaveric study. Injury 48(3):608–614 28104229 10.1016/j.injury.2017.01.028

[29] Liu ZJ, Gu Y, Jia J (2022) The Kocher-Langenbeck approach combined with robot-aided percutaneous anterior column screw fixation for transverse-oriented acetabular fractures: a retrospective study. BMC Musculoskelet Disord 23(1):345 35410204 10.1186/s12891-022-05313-w PMC8996653

[30] Chen H, Wang G, Li R et al (2016) A novel navigation template for fixation of acetabular posterior column fractures with antegrade lag screws: design and application. Int Orthop 40(4):827–834 26112873 10.1007/s00264-015-2813-8

[31] Sun Y, Chen J, Liu F, Chen Z, Li X, Lv F (2023) Study of anatomical parameters and intraoperative fluoroscopic techniques for transiliac crest anterograde lag screws fixation of the posterior column of the acetabulum. J Orthop Surg Res 18(1):697 37723587 10.1186/s13018-023-04208-3 PMC10506344

[32] Pierannunzii L, Fischer F, Tagliabue L, Calori GM, d’Imporzano M (2010) Acetabular both-column fractures: essentials of operative management. Injury 41(11):1145–1149 20828690 10.1016/j.injury.2010.08.011

[33] Baumann F, Schmitz P, Mahr D et al (2018) A guideline for placement of an infra-acetabular screw based on anatomic landmarks via an intra-pelvic approach. J Orthop Surg Res 13(1):77 29631637 10.1186/s13018-018-0786-1 PMC5892032

[34] Letournel E, Judet R (1993) Fractures of the Acetabulum. Springer, Berlin Heidelberg

[35] Arlt S, Noser H, Wienke A, Radetzki F, Hofmann GO, Mendel T (2018) Secure corridor for infraacetabular screws in acetabular fracture fixation-a 3-D radiomorphometric analysis of 124 pelvic CT datasets. J Orthop Surg Res 13(1):119 29784006 10.1186/s13018-018-0833-y PMC5963032

[36] Graul I, Marintschev I, Pizanis A, Herath SC, Pohlemann T, Fritz T (2022) The effect of an infra-acetabular screw for anatomically shaped three-dimensional plate or standard plate designs in acetabulum fractures: a biomechanical analysis. Eur J Trauma Emerg Surg 48(5):3757–3764 34618166 10.1007/s00068-021-01805-x PMC9532306

[37] Bastian JD, Näf DR, Cullmann JL, Keel MJ, Giannoudis PV (2021) Does increased acetabular depth affect safe infra-acetabular screw placement in acetabular fracture fixation? Eur J Trauma Emerg Surg 47(5):1319–1326 32728900 10.1007/s00068-020-01455-5 PMC8476395

[38] Gras F, Gottschling H, Schröder M, Marintschev I, Reimers N, Burgkart R (2015) Sex-specific differences of the infraacetabular corridor: a biomorphometric CT-based analysis on a database of 523 pelves. Clin Orthop Relat Res 473(1):361–369 25261258 10.1007/s11999-014-3932-z PMC4390952

[39] Gänsslen A, Hildebrand F, Klebingat M, Nerlich M, Lindahl J (2018) Chap. 22: special screws and views. In: Gänsslen A, Müller M, Nerlich M, Lindahl J (eds) Acetabular fractures. Georg Thieme Verlag KG, Stuttgart

[40] Dienstknecht T, Müller M, Sellei R et al (2013) Screw placement in percutaneous acetabular surgery: gender differences of anatomical landmarks in a cadaveric study. Int Orthop 37(4):673–679 23250351 10.1007/s00264-012-1740-1 PMC3609967

[41] Puchwein P, Enninghorst N, Sisak K et al (2012) Percutaneous fixation of acetabular fractures: computer-assisted determination of safe zones, angles and lengths for screw insertion. Arch Orthop Trauma Surg 132(6):805–811 22358222 10.1007/s00402-012-1486-7

[42] Shahulhameed A, Roberts CS, Pomeroy CL, Acland RD, Giannoudis PV (2010) Mapping the columns of the acetabulum–implications for percutaneous fixation. Injury 41(4):339–342 19733352 10.1016/j.injury.2009.08.004

[43] Ochs BG, Stuby FM, Stoeckle U, Gonser CE (2015) Virtual mapping of 260 three-dimensional hemipelvises to analyse gender-specific differences in minimally invasive retrograde lag screw placement in the posterior acetabular column using the anterior pelvic and midsagittal plane as reference. BMC Musculoskelet Disord 16:240 26341003 10.1186/s12891-015-0697-9 PMC4560873

[44] Lim EJ, Sakong S, Choi W et al (2022) The difference in the corridor of the antegrade posterior column screw according to the presence of pelvic dysmorphism. Injury 53(11):3774–3780 36045030 10.1016/j.injury.2022.08.056

[45] Feng X, Zhang S, Luo Q et al (2016) Definition of a safe zone for antegrade lag screw fixation of fracture of posterior column of the acetabulum by 3D technology. Injury 47(3):702–706 26867979 10.1016/j.injury.2016.01.026

[46] Boni G, Pires RE, Sanchez GT, Giordano V (2023) Antegrade posterior column screw fixation for acetabular fractures: it’s time to standardize the surgical technique. Injury 54:110579 38143145 10.1016/j.injury.2023.01.017

[47] Krappinger D, Schwendinger P, Lindtner RA (2019) Fluoroscopically guided acetabular posterior column screw fixation via an anterior approach. Oper Orthop Traumatol 31(6):503–512 31620832 10.1007/s00064-019-00631-0 PMC6879448

[48] Do Phuoc H, Nguyen Hoang P, Cao Ba H (2021) The use of a 3D-printed personalised drill guide for posterior column lag screw fixation in displaced transverse acetabular fracture: a case report. Int J Surg Case Rep 88:106503 34656924 10.1016/j.ijscr.2021.106503 PMC8521231

[49] Gras F, Marintschev I, Mendler F, Wilharm A, Mückley T, Hofmann GO (2008) [2D-fluoroscopic navigated screw osteosynthesis of acetabular fractures: a preliminary report]. Z Orthop Unfall 146(2):231–239 18404588 10.1055/s-2008-1038370

[50] Starr AJ, Reinert CM, Jones AL (1998) Percutaneous fixation of the columns of the acetabulum: a new technique. J Orthop Trauma 12(1):51–58 9447519 10.1097/00005131-199801000-00009

[51] Mouhsine E, Garofalo R, Borens O et al (2005) Percutaneous retrograde screwing for stabilisation of acetabular fractures. Injury 36(11):1330–1336 16051241 10.1016/j.injury.2004.09.016

[52] Bishop JA, Routt ML Jr. (2012) Osseous fixation pathways in pelvic and acetabular fracture surgery: osteology, radiology, and clinical applications. J Trauma Acute Care Surg 72(6):1502–1509 22695413 10.1097/TA.0b013e318246efe5

[53] Elmhiregh A, Hantouly AT, Alzoubi O, George B, Ahmadi M, Ahmed G (2024) The optimal fluoroscopic views to rule out intra-articular screw penetration during acetabular fracture fixation. Int Orthop 48(1):243–252 37855923 10.1007/s00264-023-06002-6 PMC10766808

